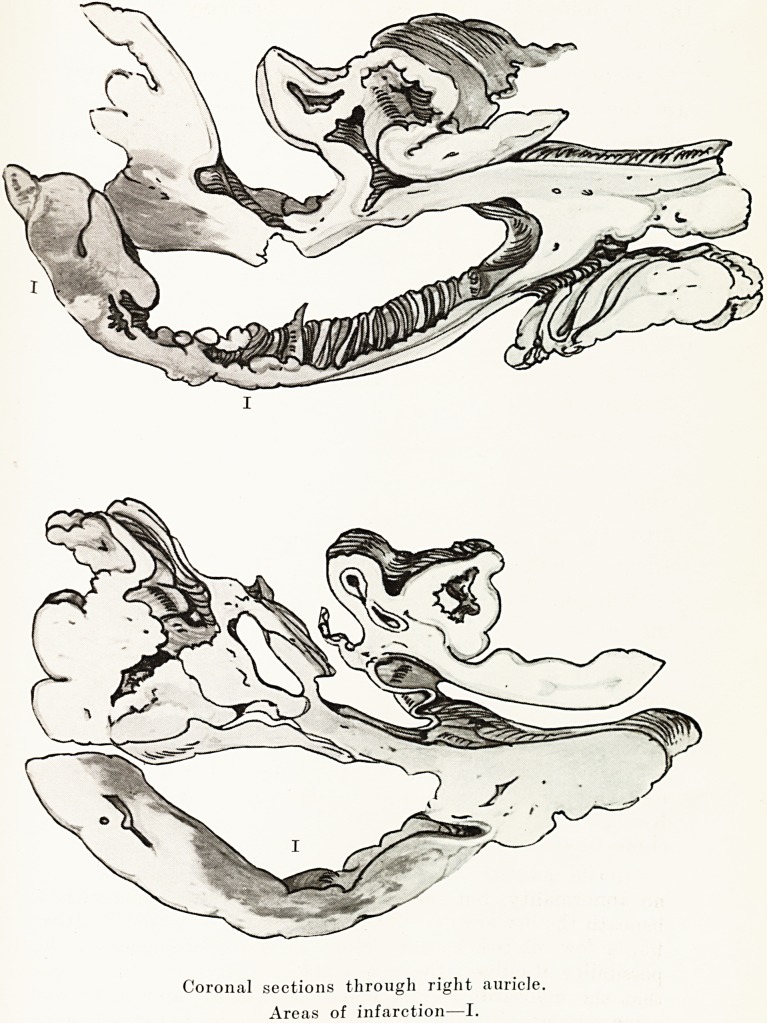# Necrosis of the Myocardium
*A Paper read at a Meeting of the Bristol Medico-Chirurgical Society, held at the University of Bristol on 10th December, 1930.


**Published:** 1931

**Authors:** Herbert Rogers

**Affiliations:** Assistant Pathologist, Bristol General Hospital, and Pathologist, Bristol Eye Hospital. (From the University Centre of Cardiac Research, Bristol General Hospital.)


### NECROSIS OF THE MYOCARDIUM.*

BY

Herbert Rogers, M.B., Ch.B.,

*Assistant Pathologist, Bristol General Hospital, and Pathologist, Bristol Eye Hospital.*

(*From the University Centre of Cardiac Research, Bristol General Hospital.*)

---

The term necrosis, if it is taken to include the death of a few cells, can be used to cover certain of the more severe degenerative changes as well as massive destruction of tissue. On this basis necrosis of the myocardium is divisible into two main groups, with certain sub-divisions.

1. *Toxic necrosis* :—
   - (A) Infective in origin.
     - (*a*) Invasion of the myocardium by organisms.
     - (*b*) Bacterial toxins causing cell death.
   - (B) Non-infective in origin.
     - (*a*) Endogenous poisons.
     - (*b*) Exogenous poisons.
2. *Ischæmic necrosis* :—
   - (*a*) Partial interference with blood supply.
   - (*b*) Complete obstruction of blood supply.

* A Paper read at a Meeting of the Bristol Medico-Chirurgical Society, held at the University of Bristol on 10th December, 1930.

In this group secondary infection may alter the histological picture of the primary ischæmic lesion.

Amongst the first group of toxic necroses there are the degenerations which occur during the course of severe anæmias, chronic diseases, and protracted febrile states. The "cloudy swelling" seen in the heart muscle is not a necrosis, as cell death is not involved, and there is little evidence that "fatty degeneration" produces anything more than a severe functional incapacity of the cell. On the other hand, in some acute infections there may be a "hyaline degeneration," in which there is coagulation of cell protoplasm, loss of striation, and destruction of the nucleus, corresponding to Zenker's degeneration of muscle seen typically in typhoid fever. This condition certainly involves cell death, and also provokes an inflammatory reaction around the affected area.

The specimens described here are illustrative of the infective and the ischæmic types of necrosis.

The first was found, post-mortem, in the heart of a boy, aged 14 years, who came into hospital on the fifth day of an acute illness, and was found to be suffering from an acute periostitis of the fifth right metacarpal bone. The signs and symptoms pointed mainly to a meningeal infection, with pyrexia, headache, vomiting, cervical rigidity, positive Kernig's sign and œdema of the optic discs. There was no clinical evidence of cardiac involvement.

At the post-mortem examination the pericardium showed no abnormality, but there were a few petechial hæmorrhages beneath the epicardium. The endocardium appeared healthy, but a few whitish specks in the myocardium suggested the possibility of miliary abscesses. Microscopically it was evident that the myocardium was acutely congested, and there was some cellular infiltration of an inflammatory nature between

### PLATE I.

Coronal sections through right auricle.
Areas of infarction—I.

the muscle cells. Further examination showed that there were minute foci of suppuration in the ventricular wall.

The second specimen showed a gross hæmorrhagic pericarditis, and a large abscess in the wall of the left ventricle which is threatening to break through the small amount of muscle left intact on either side of it. This patient was a child of 9 years of age, and the primary lesion an acute osteomyelitis of the upper end of the right humerus. Both lungs showed recent infarctions, and the liver and kidneys contained small abscesses.

In both these cases the *staphylococcus aureus* was the causal organism, and it is noticeable that when this organism gains entrance to the general circulation it tends to produce the most destructive of lesions. Thus, the endocardium may be the site of great crumbling vegetations swarming with cocci, while the myocardium may show acute necrotic foci such as are present in this case.

Such destructive lesions are very surprising when it is realized that the same organism is the usual cause of the localized skin lesions, such as boils, whitlows, impetigo, and carbuncles.

One result of these minor infections is that a relative immunity is established, so that a small number of cocci may even circulate in the blood-stream without detriment to the individual. It is when this resistance is lacking, or is temporarily lowered, or when the infection is overwhelming, that the destructive lesions within the body become possible.

The onset of pericarditis in a case of acute osteomyelitis is often looked upon as a fatal complication, but that it is not necessarily so is proved by a case recently reported from St. Bartholomew's Hospital[1] in which drainage of the pericardium was successful, and the patient, a girl of 4½ years, left the hospital convalescent after an illness of 2½ months.

The second group, that of ischæmic necrosis, has attracted considerable attention during recent years, and the case of cardiac infarction published by Dr. Carey Coombs and Professor Hadfield in 1926[2] is typical of this condition. The whole subject has been fully reviewed by Gibson[3] in this country, and more recently by Levine[4] in America. From these it appears that the necrotic area is usually limited to the ventricular walls. In this respect a case seen at the Bristol General Hospital a short time ago presented unusual features.

The patient, a man of 63 years, had complained of feeling ill for the first time three weeks before admission to the hospital. On admission he was acutely dyspnœic, perspiration was profuse, and the conjunctivæ slightly jaundiced. The temperature was 101° F., the pulse-rate 124 to 140 per minute, with a completely irregular rhythm. The blood-pressure was 110 mm. Hg. systolic, and 60 mm. Hg. diastolic. There was no œdema of the legs or back.

On examination, the area of cardiac dullness was found to be increased to the left, five inches from the middle line. The apex beat could not be palpated, and the heart sounds were weak and distant. There was dullness at both bases of the lungs, and crepitations and feeble breath sounds were noted. The abdomen and nervous system showed no abnormality.

He died on the third day after admission, and a post-mortem examination was carried out on the following day. Both lungs were very œdematous, and there were dense pleural adhesions on the left side of the chest, and also over the right upper lobe. The pericardium contained about 200 cc. of deeply blood-stained fluid with a few shreds of fibrin. The heart itself was somewhat enlarged, more especially the right side. The mitral, aortic, and the valves of the right heart appeared to be comparatively healthy, except for a small fibrous tag attached to the margin of the septal cusp of the aortic valve.

The right auricle was considerably dilated, and showed an area of acute endocarditis. An incision made into the auricular wall in this area revealed a picture closely resembling that seen in infarction of the ventricular muscle. There was a rather poorly demarcated zone of congestion or extravasation mottled with yellowish patches. The outer surface showed a definite pericarditis. The ventricular muscle presented the appearances of a moderate "fibroid myocarditis," but there was no evidence of old or recent infarction. Atheromatous changes were found in all the main branches of the coronary arteries, but no obvious obstruction or thrombosis could be demonstrated. Of the other organs the liver and the spleen were slightly enlarged and the cortical zone of the kidneys showed some reduction.

These post-mortem appearances taken in conjunction with the clinical features suggested that ischæmia of the right auricular muscle was the underlying factor and the cause of the disorder of function which resulted in death.

The blood-supply of the right auricle is derived from small vessels given off from the right coronary artery, and Dr. Perry's injection experiments show that these arterioles are very small, but numerous. Obstruction of any of these vessels in the thin auricular wall must inevitably produce damage which would involve both the pericardial and the endocardial coverings as well as the muscle itself. With chronic lung infection present, as in this case, a secondary or terminal infection of the damaged structures is almost certain.

Histologically, sections of the auricular wall showed thrombosis of the arterioles running in the musculi pectinati, and organization of the thrombus was well advanced. The muscle cells were swollen and granular, and the few remaining nuclei stained very poorly. In some areas there was considerable extravasation of blood, and in others infiltration by plasma cells and lymphocytes. The pericardial surface showed a pericarditis with a higher proportion of polymorph cells and fibrin, while the endocardium showed the changes of a subacute endocarditis. The complete picture of this case is, therefore, that of a man of 63 years whose coronary arteries are atheromatous. Thrombosis occurred in some of the arterioles supplying the right auricle, producing morbid changes which resulted in severe cardiac disorder, lowering of blood-pressure, and œdema of the lungs, and lastly a terminal infection of the damaged serous coverings of the heart.

I am greatly indebted to Dr. Carey Coombs and to Mr. John Griffiths for allowing me to use their case notes.

REFERENCES.

[1] Beattie, "Suppurative Pericarditis," *Clin. Jour.*, lviii. 522:

[2] Coombs and Hadfield, *Lancet*, 1926, i. 14.

[3] Gibson, *Ibid.*, 1925, ii. 1,270.

[4] Levine, *Medicine*, 1929, viii., No. 3, 245.

## Figures and Tables

**Figure f1:**